# Oral Administration of *Rosa gallica* Prevents UVB−Induced Skin Aging through Targeting the c−Raf Signaling Axis

**DOI:** 10.3390/antiox10111663

**Published:** 2021-10-22

**Authors:** Seongin Jo, Young-Sung Jung, Ye-Ryeong Cho, Ji-Won Seo, Won-Chul Lim, Tae-Gyu Nam, Tae-Gyu Lim, Sanguine Byun

**Affiliations:** 1 Department of Biotechnology, Yonsei University, Seoul 03722, Korea; sinnis25@naver.com (S.J.); choo9707@yonsei.ac.kr (Y.-R.C.); 2 Korea Food Research Institute, Wanju-gun 55365, Korea; chembio@khu.ac.kr (Y.-S.J.); godqhr1105@naver.com (W.-C.L.); 3 Department of Agricultural Biotechnology and Research, Institute of Agriculture and Life Sciences, Seoul National University, Seoul 08826, Korea; thinkbreaker@naver.com; 4 Major of Food Science and Biotechnology, Division of Bio-Convergence, Kyonggi University, Suwon 16227, Korea; tgzoo0706@kyonggi.ac.kr; 5 Department of Food Science & Biotechnology, Sejong University, Seoul 05006, Korea

**Keywords:** *Rosa gallica*, skin aging, gallic acid, c−Raf, UHPLC−MS/MS

## Abstract

*Rosa gallica* is a widely used Rosa species for medicinal and culinary purposes. *Rosa gallica* has been reported to display antioxidant, anti−inflammatory, and antibacterial activities. However, the effect of *Rosa gallica* against skin aging in vivo is unknown and its active components have not been fully understood. Oral administration of *Rosa gallica* prevented UVB−mediated skin wrinkle formation and loss of collagen/keratin fibers in the dorsal skin of mice. Examination of biomarkers at the molecular level showed that *Rosa gallica* downregulates UVB−induced COX−2 and MMP−1 expression in the skin. Through a direct comparison of major compounds identified using the UHPLC−MS/MS system, we discovered gallic acid as the primary component contributing to the anti-skin aging effect exhibited by *Rosa gallica*. Examination of the molecular mechanism revealed that gallic acid can potently and selectively target the c−Raf/MEK/ERK/c−Fos signaling axis. In addition, both gallic acid and MEK inhibitor blocked UVB−induced MMP−1 expression and restored collagen levels in a reconstructed 3D human skin model. Collectively, *Rosa gallica* could be used as a functional ingredient in the development of nutraceuticals against skin aging.

## 1. Introduction

Chronic irradiation of ultraviolet B (UVB) light causes skin wrinkle formation, inflammation, pigmentation, and dehydration [1,2]. UVB triggers signaling pathways leading to upregulation of genes involved in collagen degradation and inflammation [1,3]. Among these, matrix metalloproteinases (MMPs), especially MMP−1, play a key role in promoting collagen degradation and in turn, wrinkle formation [4]. In addition, cyclooxygenase 2 (COX−2), which can be induced by UVB light, mediates skin inflammation and photoaging [5]. Thus, food compounds that can inhibit the expression of MMP−1 or/and COX−2 have the potential to possess anti-skin aging effects [6,7].

Activator protein 1 (AP−1) and its upstream regulatory pathways have been known to be major contributing factors to MMP–1 expression and skin aging [8]. AP−1 is a dimeric transcription factor formed by Fos (c−Fos, FosB, Fra1, and Fra2) and Jun (c−Jun, JunB, and JunD) families. Activation of AP−1 induces transcription of genes involved in skin aging, cell proliferation, angiogenesis, and inflammation [8,9,10]. Especially as AP−1 directly induces MMP−1 expression leading to collagen degradation in the skin, downregulation of AP−1 components have been suggested as a promising strategy to block skin aging [11]. UV causes the activation of mitogen−activated protein kinases (MAPKs), including ERK1/2, p38, and JNK1/2, which act as key upstream regulators of AP−1 activity [8,12]. Hence, studies have demonstrated that inhibiting upstream regulatory pathways of AP−1, such as the MAPKs, MAP2Ks, or c−Raf can suppress skin aging [13,14,15,16,17].

Rosa species have been used as a cooking ingredient as well as a medicinal plant. Various types of roses, including *Rosa canina*, *Rosa damascene*, *Rosa centifolia*, and *Rosa gallica* have been reported to display antioxidant, antibacterial, anti−depressant, and anti−inflammatory properties [18,19]. Additionally, the Rosa species is known for its functional effects on the skin [20,21]. In particular, *Rosa gallica* is one of the most commonly used Rosa species for cosmetic, medicinal, and culinary purposes [22,23]. Although the potential of the *Rosa gallica* extract to prevent biomarkers of skin aging in vitro has been reported [24], there has been no study demonstrating the anti−skin aging effect of *Rosa gallica* in vivo. Moreover, active ingredients responsible for the bioactivity of *Rosa gallica* and Rosa species are poorly understood. Herein, we have orally administered the *Rosa gallica* extract to mice and evaluated its anti−skin aging effect to assess its potential as a functional food/nutraceutical agent. Moreover, we have identified the active ingredient of *Rosa gallica* by ultra−high performance liquid chromatography (UHPLC) with mass spectrometry (MS) and examined its molecular mechanism.

## 2. Materials and Methods

### 2.1. Materials

Gallic acid, quercetin, catechin, kaempferol, rutin, quercitrin, U0126, dimethyl sulfoxide (DMSO), and formic acid were purchased from Sigma−Aldrich (St. Louis, MO, USA). Antibody to detect COX−2 was purchased from Cayman Chemical (Ann Arbor, MI, USA), and the antibodies to detect MMP−1 and collagen Ι were obtained from Abcam (Cambridge, United Kingdom). Antibodies against c−Jun, p38, JNK, c−Raf, MKK4, vinculin, and GAPDH was provided by Santa Cruz Biotechnology, Inc. (Dallas, TX, USA). Antibody to detect c−Fos, phospho−c−Jun, phospho−ERK, ERK, phospho−p38, phospho−JNK, phospho−c−Raf, phospho−MEK, MEK, phospho−MKK4, phospho−MKK3, and MKK3 were purchased from Cell Signaling Technology (Danvers, MA, USA). *Rosa gallica* petals were imported from Turkey through GN Bio (Hanam, Korea). Analytical grade water and acetonitrile (ACN) were purchased from Thermo Fisher Scientific (Waltham, MA, USA).

### 2.2. Rosa gallica Extract Preparation

Rose petals were ground in a blender to obtain a powder. Dried powder (10 g) of rose petal was mixed with 1000 mL of 70% (*v*/*v*) ethanol and extracted at 70 ℃ for 3 h using reflux condenser. And then the extract was filtered through No. 2 filter paper (Whatman, Maidstone, UK). The solvent was subsequently evaporated, and the product was freeze-dried.

### 2.3. Experimental Animals and Treatments

Female SKH−1 hairless mice were obtained from Orient Bio (Seongnam, Korea). Mice had free access to food and water for 15 weeks. Seven mice were allocated into each group. The Institutional Animal Care and Use Committee (SEMI1−19−02) approved all experimental protocols. *Rosa gallica* extract was dissolved in distilled water at the indicated concentration and treated to mice every day by oral gavage. UVB was applied to the mice by UV−3000 (Dong Seo Science Co., Ltd., Seoul, Korea) starting at week 5 of the experiment. UVB irradiation was gradually increased from 1 MED to 4 MED (1 MED = 1 minimal erythema dose = 50 mJ/cm^2^) with no injury.

### 2.4. Wrinkle Measurement

Skin replicas of mouse dorsal skin were made with the SILFLO (Amique Group Co. Ltd., Tokyo, Japan) at the end of the experiments. The skin replicas were photographed using Nikon E600 (Nikon, Tokyo, Japan). Depth of the wrinkle was measured by Visioline^®^ VL 650 (Courage&Khazaka GmbH, Koln, Germany).

### 2.5. Masson’s Trichrome Staining

Tissue specimens were fixed with 4% (*v*/*v*) formalin solution. Tissue was embedded in paraffin. Approximately 4 μm−thick sections were subjected to stained with Masson’s Trichrome for collagen and keratin fibers analysis. After tissue staining, slides were examined at 200 X magnification by Nikon DS–Fi3 (Nikon).

### 2.6. Immunoblot

Skin tissues or cell were lysed with RIPA buffer. Immunoblot was performed as previously described [25]. Briefly, lysates were centrifuged at 4 °C, 12,000× *g* for 10–20 min. Protein concentration of lysate was measured using BCA assay (Thermo Fisher Scientific). The proteins were separated using SDS−PAGE and transferred to a nitrocellulose (NC) membrane (PALL^®^ Corporation, Port Washington, NY, USA). The NC membrane was blocked in the 5% skim milk in Tris−Buffered Saline in 0.1% Tween 20 for 1 hr. Membrane was incubated with a primary antibody at 4 °C overnight. Bands were detected with Western Lightning Plus−ECL (PerkinElmer, Waltham, MA, USA) after incubated with an HRP−conjugated secondary antibody. All blots presented in the manuscript are from a film scan generated from an automatic X−ray film processor (JPI Healthcare, Seoul, Korea).

### 2.7. UHPLC–LTQ–Orbitrap/MS/MS Conditions

The molecular weights of the peaks were displayed from high−resolution MS using LTQ Orbitrap XL (Thermo Fisher Scientific) coupled with Accelar UHPLC (Thermo Fisher Scientific). Mobile phase A was water containing 0.1% (*v*/*v*) formic acid, and mobile phase B was ACN containing 0.1% (*v*/*v*) formic acid. Separation of compounds was performed using a C_18_ column (Acquity UPLC^®^ BEH; 2.1 mm × 100 mm, 1.7 μm; Waters Corp., Milford, MA, USA) at a flow rate of 0.4 mL/min using the following gradient 3–10% mobile phase B for 3 min; 10–40% B for 16 min; 40–80% B for 17 min; 80–3% B for 19 min; 3% B for 20 min. The UHPLC−MS/MS was operated with a Z−spray ion source in the positive ion, negative ion mode using the following conditions: capillary voltage of 20 V, capillary temperature of 350 ℃, spray voltage 3.5 kV. Spectra were scanned in the range between 150 and 1500 *m/z*.

### 2.8. Cell Culture and UVB Irradiation

Human Dermal fibroblasts (HDFs) were kindly provided by Dr. Jin Ho Chung (Department of Dermatology, Seoul National University College of Medicine, Seoul, Korea). HDFs were cultured in Dulbecco’s Modified Eagle’s Media (DMEM, Corning Inc., Somerville, MA, USA) containing 10% fetal bovine serum (FBS, Thermo Fisher Scientific) with penicillin/streptomycin (Corning Inc.). Cells were irradiated with UVB using Bio−link−BLX (Vilber Lourmat, Paris, France), with the peak emission wavelength is 312 nm. The energy of UV was 0.03 J/cm^2^.

### 2.9. SRB Staining

HDFs were seeded into a 6−well plate. After 24 h, the medium was replaced with serum–free DMEM. The next day, chemicals were treated at the indicated concentrations and incubated for 48 h. After fixing the cells, the living cells were stained with sulforhodamine B (SRB, Sigma−Aldrich). SRB was dissolved with 10 mM Tris and absorbance was measured at 554 nm.

### 2.10. Enzyme-Linked Immunosorbent Assay (ELISA)

HDFs were seeded in 12−well plates at a density of 1.8 × 10^5^ cells/well. Cells were incubated for 24 h, after which the medium was replaced with serum−free media and incubated for another 24 h. Cells were treated with compounds at the indicated concentrations and then irradiated with UVB. Following 48 h of incubation, the culture supernatants were collected and centrifuged at 13,000× *g* to remove cell debris. The concentration of MMP−1 in the cell culture media was determined by corresponding ELISA kits (R&D Systems Inc., Minneapolis, MN, USA). These assays were performed as manufacturer’s instructions.

### 2.11. Reconstructed 3D Human Skin Model

Reconstructed 3D human skin (Neoderm−ED) was obtained from Tegoscience (Seoul, Korea). The reconstructed 3D human skin model was treated with indicated chemicals for 1 h prior to UVB irradiation. The skin tissue was irradiated with UVB twice a day for 8 days, and the medium was changed every two days. The skin tissue was incubated at 37 °C under 5% CO_2_ atmosphere.

### 2.12. Collagen Staining in Reconstructed 3D Human Skin Model

Skin sections from reconstructed 3D human skin model were fixed with 4% formalin solution. Paraffin−embedded sections were cut on glass slides. Slides were deparaffinized three times with xylene and hydrated through a grade alcohol bath. The deparaffinized sections were stained with Sirius Red/Fast Green solution (Chondrex, Inc. Woodinville, WA, USA). After tissue staining, slides were washed with 0.5% (*v*/*v*) acetic acid solution. Finally, slides were dehydrated through a reverse grade alcohol bath and mounted. Pictures of collagen−stained slides were taken using the Nikon eclipse Ts2 (Nikon).

### 2.13. Statistical Analysis

Statistical analyses were used one−way analysis of variance followed by GraphPad Prism 5 software (San Diego, CA, USA). All data are presented as means ± standard deviation (SD). A *p* < 0.05 was considered statistically significant.

## 3. Results

### 3.1. Oral Consumption of Rosa gallica Petal Extract (RPE) Suppresses UVB−Mediated Skin Wrinkle In Vivo

**Figure 1:**
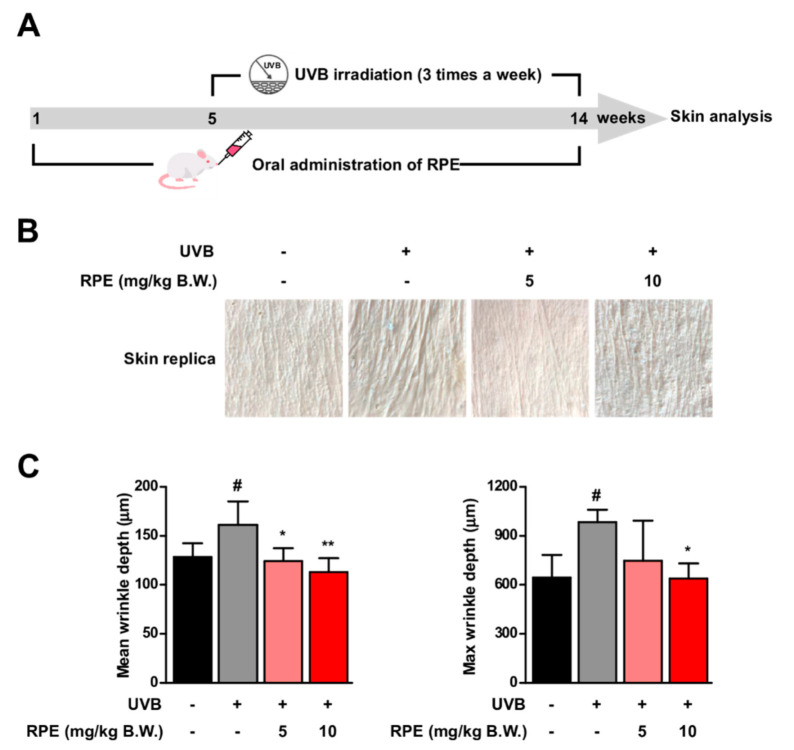
Rose petal extract (RPE) suppresses UVB−induced skin wrinkle formation. (**A**) RPE was orally administered to mice every day. UVB was irradiated three times a week as indicated in the experimental scheme. (**B**) Representative pictures of skin replica. (**C**) Mean wrinkle depth and max wrinkle depth were quantified. Data represent the means ± SD (*n* = 4). Significant differences between un−treated control and UVB−only treatment group (# *p* < 0.05) and significant differences between UVB and UVB + RPE administrated group (* *p* < 0.05, ** *p* < 0.01).

Mice were administered with *Rosa gallica* petal extract (RPE) and exposed to UVB for 10 weeks (Figure 1A). Continuous exposure to UVB induced skin wrinkle formation (Figure 1B). Interestingly, oral administration of RPE at 5 and 10 mg/kg B.W. led to a reduction in UVB−mediated wrinkle formation in the dorsal skin of mice (Figure 1B). Quantification of skin wrinkle demonstrated that RPE administration could block UVB−induced mean wrinkle depth and max wrinkle depth to near control levels (Figure 1C). These results clearly show that oral intake of RPE can suppress UVB−mediated skin aging in vivo.

### 3.2. RPE Does Not Show Side Effects In Vivo

**Figure 2:**
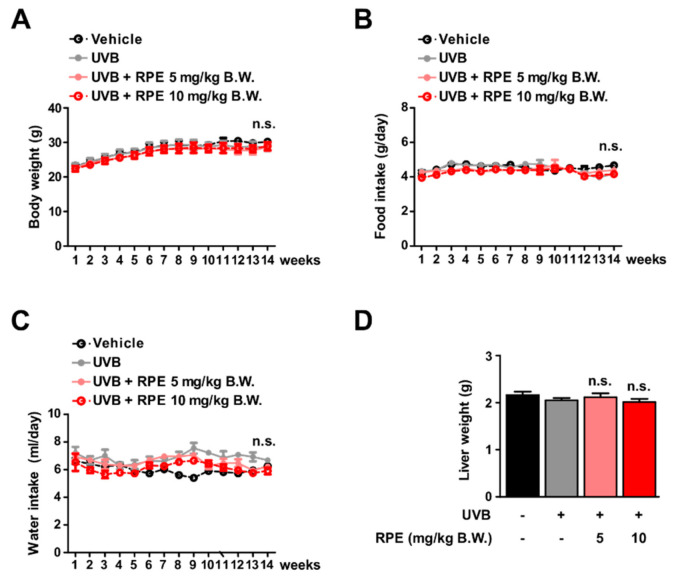
RPE does not show toxicity at the tested concentrations. (**A**–**C**) Body weight (*n* = 7), food intake (*n* = 4), and water intake (*n* = 4) were measured twice a week. (**D**) Liver weight was measured after sacrificing the mice at the end of the experiment (*n* = 7). Data represent the means ± SD. There was no significant difference (n.s.) among all groups.

To assess the safety of RPE in vivo, we evaluated several parameters after RPE administration. RPE did not display noticeable effects on body weight of mice (Figure 2A). In addition, RPE did not affect the amount of food and water intake (Figure 2B,C). Additionally, RPE did not cause any change to the weight of liver in the mice (Figure 2D). Collectively, these results demonstrate that oral consumption of RPE does not generate side effects in vivo at the treated concentrations.

### 3.3. RPE Inhibits Collagen Degradation and Blocks UVB−Mediated Biomarker of Skin Aging

**Figure 3:**
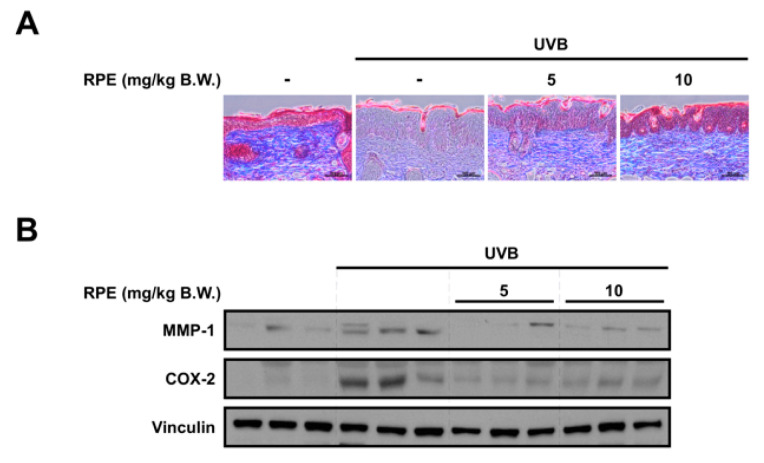
RPE inhibits collagen degradation and reverses biomarkers of skin aging. (**A**) Masson’s trichrome staining for the visualization of collagen and keratin fibers in mouse skin tissue. Collagen fibers appear blue and keratin fibers appear red. The scale bar indicates 100 μm. (**B**) Protein expression of MMP−1, COX−2, and vinculin were determined in mouse tissue lysates using the corresponding antibody. Tissue lysates from three mice per group were used. Vinculin was used as a loading control.

To further examine the anti−skin aging effect of RPE, we analyzed key biomarkers of skin aging. Collagen and keratin are major structural proteins constituting the skin and reduction of these proteins has been known to be responsible for skin wrinkle formation [26,27]. Results from Masson’s trichrome staining of the skin demonstrate that RPE can prevent UVB−mediated decrease in collagen and keratin levels in the skin (Figure 3A). We also found that RPE suppresses UVB−induced COX−2 and MMP−1 expression in the skin (Figure 3B), suggesting that RPE can block major mediators of inflammation and collagen degradation. While there has been a previous report that RPE can attenuate MMP−1 in cells, this is the first time to show that RPE can suppress MMP−1 expression as well as collagen degradation and wrinkle formation in vivo.

### 3.4. Identification of Compounds in Rosa gallica

**Figure 4:**
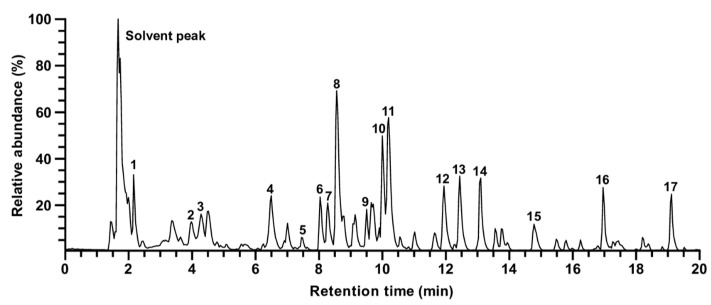
The peak intensity chromatogram of UHPLC−LTQ−Orbitrap/MS/MS of RPE. Peaks are indicated in Table 1.

Main fragment ions of RPE obtained by UHPLC–LTQ–Orbitrap/MS/MS are shown in Figure 4. A total of 17 peaks excluding solvent peak were identified (Table 1). Tentative identification was performed using the obtained precursor ion and their fragment ions. Gallic acid (1) and catechin (2), which are relatively polar compounds, were detected rapidly with short retention times under reverse−phase condition. Compounds in which two glycosides are bound to quercetin (such as rutin, fragment ion value of 303.05 *m*/*z*) were identified at relatively short retention times, followed by a monoglycoside (quercetin of 303.05 *m*/*z* and kaempferol of 287.05 *m*/*z*) and an aglycone (dihydrokaempferol) type. In this study, since the binding structures of the glycoside moiety is unclear, it was expressed as a diglycoside. Among the identified compounds, 11 peaks were identified as quercetin (peaks no. 5, 6, 7, 8, 11, 14, and 15) or kaempferol (peaks no. 9, 10, 13, and 17) derivatives, which is in accordance with previous reports where the glycosides of quercetin and kaempferol are the most abundant flavonoids in roses among the reported compounds [28,29,30].

### 3.5. Gallic Acid Is a Major Active Compound of Rosa gallica in Preventing Skin Aging

**Figure 5:**
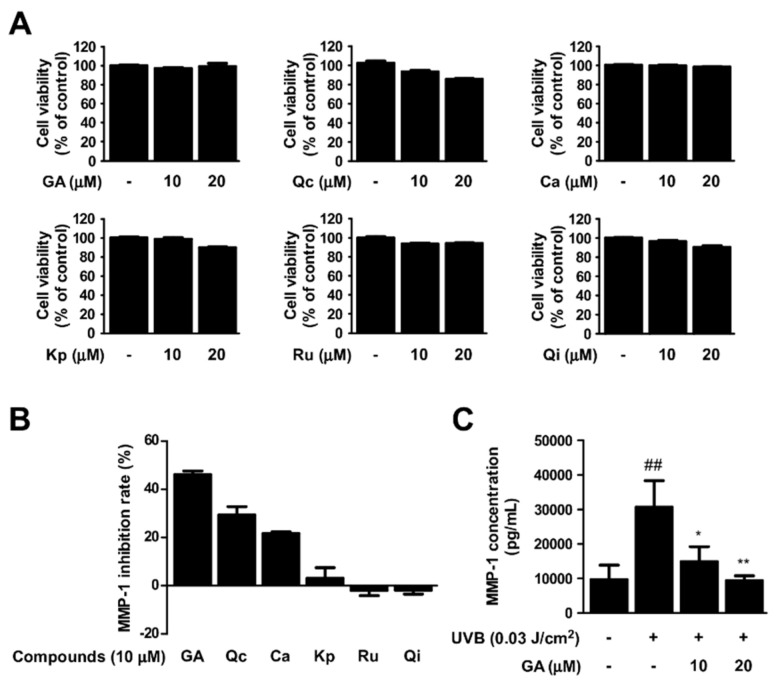
Effect of single compounds in RPE on UVB−induced MMP−1. (**A**) Cell viability was measured after cells were treated with gallic acid (GA), quercetin (Qc), catechin (Ca), kaempferol (Kp), rutin (Ru), Quercitrin (Qi) for 48 h (*n* = 5). (**B**) Human dermal fibroblasts (HDFs) were pre−treated with compounds at the indicated concentrations for 1 h before being exposed to UVB. After 48 h, MMP−1 production in cultured media was measured using ELISA (*n* = 3). Data represent MMP−1 inhibition rate compared to UVB−only treatment group. (**C**) HDFs were pre−treated with gallic acid (GA). MMP−1 concentration was measured using ELISA (*n* = 3). Data represent the means ± SD. Significant differences between un−treated control and UVB−only treatment group (## *p* < 0.01) and significant differences between UVB and UVB + GA treatment group (* *p* < 0.05, ** *p* < 0.01).

Based on the results from the chemical analysis (Figure 4 and Table 1), we selected gallic acid, quercetin, catechin, kaempferol, rutin, and quercitrin for further evaluation. The inhibitory activity against MMP−1 was measured using the six compounds. Treatment of gallic acid displayed the strongest reduction in UVB−induced MMP−1 levels among the tested compounds without any apparent cytotoxicity (Figure 5A,B). In addition, gallic acid was able to suppress MMP−1 levels in a dose−dependent manner (Figure 5C), suggesting gallic acid as a major contributing factor to the anti−skin aging effect exhibited by RPE.

### 3.6. Gallic Acid Targets the c−Raf Signaling Pathway

**Figure 6:**
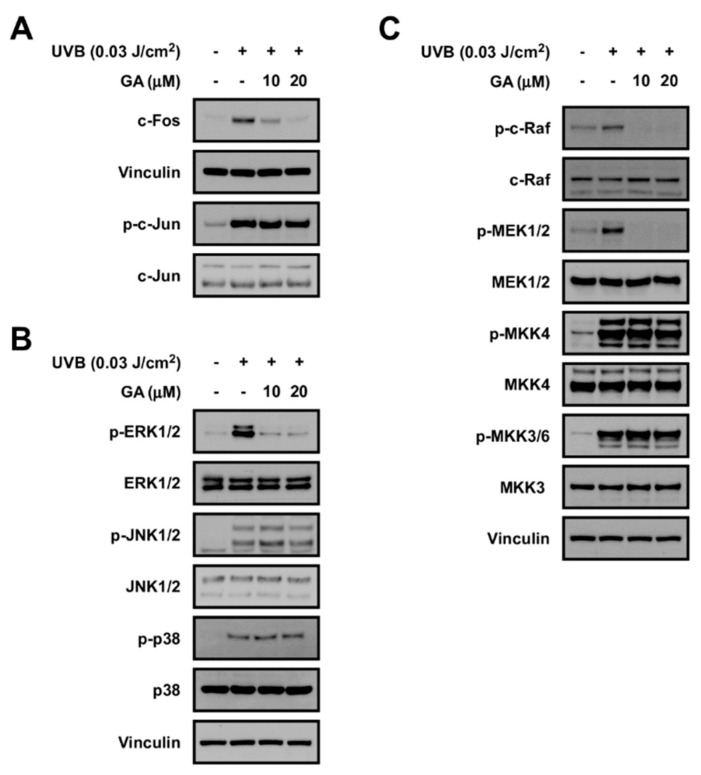
Effect of gallic acid (GA) on UVB−induced signaling pathways. (**A**) Protein expression of c−Fos, vinculin, p−c−Jun, and c−Jun were determined in human fibroblast cell lysates using the corresponding antibody. Vinculin was used as a loading control. (**B**) Effect of GA on MAPKs pathway in HDFs. Protein expression levels of phosphorylated and total ERK, JNK, p38, and vinculin were determined in cell lysates using the corresponding antibody by immunoblotting. Vinculin was used as a loading control. (**C**) Effect of GA on c−Raf and MAP2Ks pathway in HDFs. Protein expression levels of phosphorylated and total c−Raf, MEK1/2, MKK4, and MKK3, and vinculin were determined in cell lysates using the corresponding antibody by immunoblotting. Vinculin was used as a loading control.

To understand the molecular mechanism of gallic acid, we investigated the effect of gallic acid on UVB−mediated signaling. As AP−1 is a crucial transcription factor controlling the expression of MMP−1 [4], we sought the test the effect of gallic acid on individual factors constituting the AP−1 dimer. Gallic acid downregulated c−Fos, whereas phosphorylation of c−Jun was not affected (Figure 6A). Since the expression of c−Fos has been known to be primarily regulated by MAPK family members (i.e., ERK1/2, p38, and JNK1/2) [8], we examined the effect of gallic acid against MAPK activation. We discovered that gallic acid can potently and selectively suppress ERK1/2 phosphorylation, while displaying no noticeable effects on JNK1/2 and p38 phosphorylations (Figure 6B). Gallic acid also attenuated phosphorylation of MEK1/2 which is the direct upstream regulator of ERK1/2 (Figure 6C). c−Raf is activated by UV and acts as an upstream regulator of the MEK/ERK pathway [36]. Results show that gallic acid can markedly reduce the activation of c−Raf (Figure 6C). In addition, gallic acid did not affect the phosphorylations of MKK4 and MKK3/6, further suggesting that gallic acid preferentially inhibits the c−Raf/MEK pathway (Figure 6C). Overall, these results show that gallic acid can selectively target the c−Raf/MEK/ERK/c−Fos pathway.

### 3.7. MEK Inhibition and Gallic Acid Blocks UVB−Induced MMP−1 Expression and Collagen Reduction in Reconstructed 3D Human Skin Model

**Figure 7:**
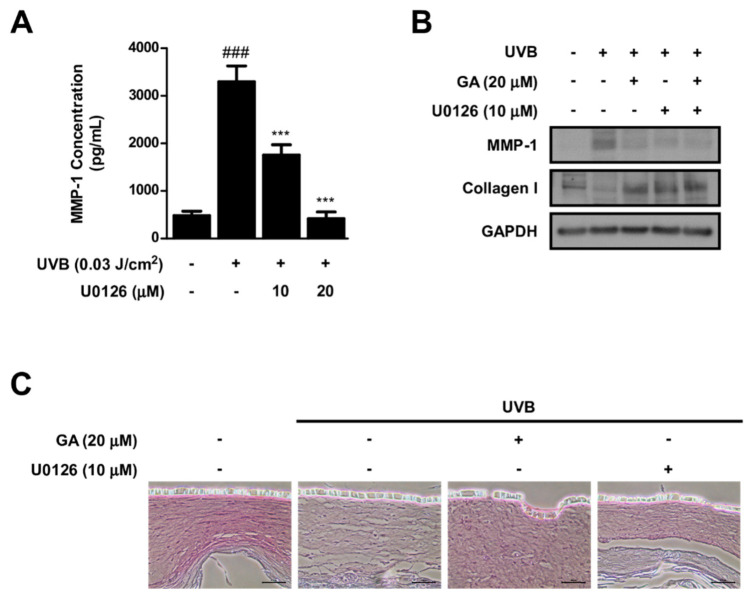
Effect of U0126 and gallic acid on MMP−1 and collagen expression in reconstructed 3D human skin model. (**A**) HDFs were pre−treated with U0126. MMP−1 concentration was measured using ELISA (*n* = 3). Data represent the means ± SD. Significant differences between un−treated control and UVB−only treatment group (### *p* < 0.001) and significant differences between UVB and UVB + U0126 treatment group (*** *p* < 0.001). (**B**) Effect of GA and U0126 on MMP−1 and collagen in a reconstructed 3D human skin model. GAPDH was used as a loading control. (**C**) Collagen was stained in sections from a reconstructed 3D human skin model using Sirius Red/Fast Green solution. The scale bar indicates 50 μm.

As gallic acid selectively inhibited the c−Raf/MER/ERK signaling axis, we used a selective MEK inhibitor (i.e., U0126) to confirm the involvement of this signaling pathway in MMP−1 and collagen expression. U0126 suppressed UVB−induced MMP−1 in HDFs, demonstrating that activation of c−Raf/MER/ERK signaling is necessary for MMP−1 production (Figure 7A). We further utilized a reconstructed 3D human skin model to investigate the impact of U0126 and gallic acid on controlling MMP−1 and collagen levels. Reconstructed 3D human skin tissues were treated with U0126, gallic acid, or both and irradiated with UVB for 8 days. Both gallic acid and U0126 downregulated UVB−induced MMP−1 expression and prevented UVB−induced decrease in collagen (Figure 7B,C).

## 4. Discussion

In the current study, RPE was orally administered to mice and its protective effect against skin aging was examined. While a previous study reported the effect of RPE against UVB−induced skin aging under in vitro conditions [24,37], the efficacy of RPE in vivo was unknown. This is the first report to demonstrate that oral administration of *Rosa gallica* can block UVB−mediated skin aging in vivo without any noticeable toxicity. These results suggest that RPE can be a promising nutraceutical agent for the prevention of skin aging.

While previous studies reported bioactivities of *Rosa gallica*, the active components remained elusive. The identification of active compounds is a crucial step in understanding the mechanism of natural agents as well as developing it for practical applications. The active compound can be used as a marker for quality control or for optimizing processing conditions of the natural agent. Through combining UHPLC−MS/MS−based chemical analysis and activity evaluation, we have identified gallic acid as an active compound that can at least partially recapitulate the anti−skin aging effect exerted by RPE. Gallic acid was chosen as the major active compound because it showed the strongest inhibitory effect against MMP−1 expression compared to other compounds from RPE. Although weaker than gallic acid, quercetin and catechin also displayed inhibitory activity against MMP−1 expression (Figure 5B). This is consistent with previous studies which have also reported the anti-skin aging potential of quercetin and catechin [38,39]. Thus, the protective effect of RPE against skin aging may be attributed to a combination of several components. Based on our results gallic acid was chosen as a major active component, yet further studies to evaluate the activity of other compounds could aid in fully understanding the function of *Rosa gallica*.

When we examined the components of RPE, glycosidic derivatives of quercetin and kaempferol constituted a large portion among the identified compounds (Table 1). Rutin and quercitrin showed significantly weaker bioactivity compared to their aglycon compound, quercetin (Figure 5B), which is in accordance with previous reports where removal of the sugar moieties generally increases the bioactivity of the compound [40,41,42,43]. In line with this, while we were not able to test all the compounds found in the extract, considering that kaempferol generated minor effects in the tested condition (Figure 5B), it is likely that other glycosidic derivatives of kaempferol found in RPE would also produce relatively insignificant effects towards MMP−1 expression. However, there is a possibility that other compounds with distinct structures found in RPE could contribute to the anti-skin aging effect of *Rosa gallica*.

We have examined the molecular mechanism of gallic acid and discovered that gallic acid can selectively inhibit c−Raf, MEK, ERK, and c−Fos, while displaying no noticeable effects towards other MAPK and MAP2K family members. Gallic acid was previously reported to exert anti−skin aging effects; however, the responsible mode of action was largely unknown [44,45]. We report that gallic acid can inhibit UVB−induced c−Raf/MEK/ERK/c−Fos signaling axis in HDFs, and this may be the major mechanism to explain the gallic acid−driven downregulation of MMP−1. In addition, the c–Raf/MEK/ERK signaling pathway has been known to play a critical role in the development of various cancers, including melanoma, non−melanoma skin cancers, pancreatic cancer, and non−small cell lung cancer [46,47,48]. Considering that agents targeting the c−Raf pathway exert chemopreventive/chemotherapeutic effects against these types of cancers [47,49], gallic acid may also potentially suppress carcinogenesis.

At high concentrations phytochemicals may display toxicity, however, at low concentrations, they can modulate various physiological pathways, potentially providing health benefits. The concept of hormesis has been applied to understand the mechanism for the therapeutic effects reported by natural products [50,51]. Among these, the activation of cellular stress response pathways has been suggested as one of the potential modes of action for phytochemicals [51]. Indeed, many phytochemicals have been implicated to control cellular antioxidant systems as well as the signal transduction pathways [51,52]. In addition to targeting the c−Raf/MEK/ERK/c−Fos signaling axis, *Rosa gallica* and gallic acid may have exerted anti-skin aging activity through affecting regulators of oxidative stress. Gallic acid has been reported to modulate Keap1/Nrf2/ARE pathway [53,54] and Roses have been known to show antioxidant effects in cell models [37,55]. These attributes of *Rosa gallica* and gallic acid could have provided cellular protection through regulating the concentration of free radicals and subsequent inflammatory responses in the skin, leading to attenuation of skin aging.

## 5. Conclusions

**Figure 8:**
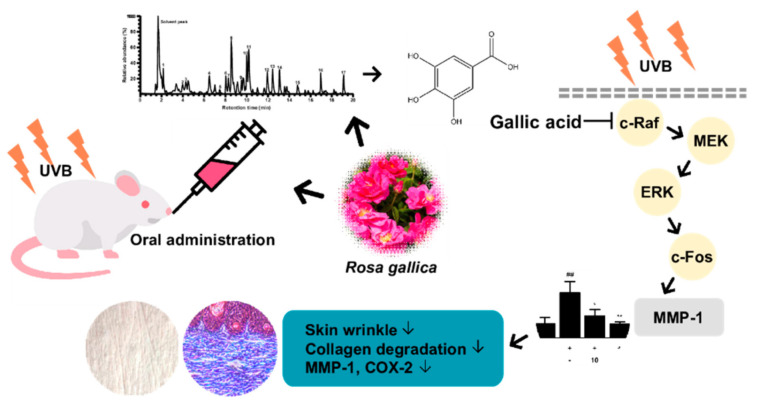
Schematic diagram summarizing the anti−skin aging effect of *Rosa gallica* and its mechanism.

*Rosa gallica* is an edible flower which has been used as an ingredient in traditional medicine and culinary practices. We have found that *Rosa gallica* can provide a protective function against skin aging in vivo. Gallic acid appears to function as an active compound of *Rosa gallica* through inhibiting the c−Raf signaling pathway (Figure 8). Discovering the possibility of *Rosa gallica* as an anti−skin aging food agent opens new opportunities to utilize rose petals as a nutraceutical.

## Figures and Tables

**Table 1:** Identification of major chemical constituents in RPE by UHPLC−LTQ−Orbitrap/MS/MS.

Peak	t_R_ (min)	Molecular Weight	Ion Mode	Precursor Ion (*m/z*)	Fragment Ions (*m/z*)	Tentative Identification	Reference
1	2.16	170.1195	[M − H]^−^	169.0000	N.D.	Gallic acid	[30]
2	4.04	290.2681	[M + H]^+^	291.0864	N.D.	Catechin	[29]
3	4.28	954.7038	[M + H]^+^	955.1054	N.D.	Rugosin B	[28]
4	6.50	936.6454	[M + H]^+^	937.0934	N.D.	Casuarictin	[31]
5	7.45	616.4806	[M + H]^+^	617.1137	303.0499	Quercetin-3-*O*-gallate-glucoside	[32]
6	8.00	610.5175	[M + H]^+^	611.1607	465.1029, 303.0500	Rutin	[30]
7	8.28	464.3763	[M + H]^+^	465.1028	303.0499	Quercetin-3-*O*-galactoside	[28,33]
8	8.56	464.3763	[M + H]^+^	465.1026	303.0498	Quercetin-3-*O*-glucoside	[30,33]
9	9.51	448.3769	[M + H]^+^	449.1078	287.0549	Kaempferol-3-*O*-galactoside	[28,33]
10	10.00	448.3769	[M + H]^+^	449.1080	287.0550	Kaempferol-3-*O*-glucoside	[30,33]
11	10.20	448.3769	[M + H]^+^	449.1078	303.0498	Quercitrin	[30]
12	11.94	438.5128	[M − H]^−^	437.1445	N.D.	unknown	
13	12.44	432.3775	[M + H]^+^	433.1130	287.0550	Kaempferol-3-*O*-rhamnoside	[30]
14	13.10	652.5542	[M + H]^+^	653.1712	303.0500	Quercetin diglycoside	[30]
15	14.78	636.5548	[M + H]^+^	637.1764	303.0500	Quercetin diglycoside	[30]
16	16.96	583.6741	[M + H]^+^	584.2754	438.2387	Tricoumaroyl spermidine	[34]
17	19.12	288.2522	[M + H]^+^	289.2373	271.2268	Dihydrokaempferol	[35]

RPE, rose petal extract; UHPLC-LTQ-Orbitrap/MS/MS, ultra-high performance liquid chromatography and tandem mass spectrometry; t_R_, retention time; N.D., not detected.

## Data Availability

Data is contained within the article.

## References

[1] Yaar M., Gilchrest B.A. (2007). Photoageing: Mechanism, prevention and therapy. Br. J. Derm..

[2] Perez-Sanchez A., Barrajon-Catalan E., Herranz-Lopez M., Micol V. (2018). Nutraceuticals for Skin Care: A Comprehensive Review of Human Clinical Studies. Nutrients.

[3] Rittie L., Fisher G.J. (2015). Natural and sun-induced aging of human skin. Cold Spring Harb. Perspect. Med..

[4] Fisher G.J., Kang S., Varani J., Bata-Csorgo Z., Wan Y., Datta S., Voorhees J.J. (2002). Mechanisms of photoaging and chronological skin aging. Arch. Derm..

[5] Surowiak P., Gansukh T., Donizy P., Halon A., Rybak Z. (2014). Increase in cyclooxygenase-2 (COX-2) expression in keratinocytes and dermal fibroblasts in photoaged skin. J. Cosmet Derm..

[6] Philips N., Auler S., Hugo R., Gonzalez S. (2011). Beneficial regulation of matrix metalloproteinases for skin health. Enzym. Res..

[7] Habib M.A., Salem S.A., Hakim S.A., Shalan Y.A. (2014). Comparative immunohistochemical assessment of cutaneous cyclooxygenase-2 enzyme expression in chronological aging and photoaging. Photodermatol. Photoimmunol. Photomed..

[8] Rittie L., Fisher G.J. (2002). UV-light-induced signal cascades and skin aging. Ageing Res. Rev..

[9] Angel P., Szabowski A., Schorpp-Kistner M. (2001). Function and regulation of AP-1 subunits in skin physiology and pathology. Oncogene.

[10] Li Y., Alhendi A.M.N., Yeh M.C., Elahy M., Santiago F.S., Deshpande N.P., Wu B., Chan E., Inam S., Prado-Lourenco L. (2020). Thermostable small-molecule inhibitor of angiogenesis and vascular permeability that suppresses a pERK-FosB/DeltaFosB-VCAM-1 axis. Sci Adv..

[11] Pittayapruek P., Meephansan J., Prapapan O., Komine M., Ohtsuki M. (2016). Role of Matrix Metalloproteinases in Photoaging and Photocarcinogenesis. Int. J. Mol. Sci..

[12] Price M.A., Cruzalegui F.H., Treisman R. (1996). The p38 and ERK MAP kinase pathways cooperate to activate Ternary Complex Factors and c-fos transcription in response to UV light. EMBO J..

[13] De Araujo R., Lobo M., Trindade K., Silva D.F., Pereira N. (2019). Fibroblast Growth Factors: A Controlling Mechanism of Skin Aging. Ski. Pharm. Physiol.

[14] Radler-Pohl A., Sachsenmaier C., Gebel S., Auer H.P., Bruder J.T., Rapp U., Angel P., Rahmsdorf H.J., Herrlich P. (1993). UV-induced activation of AP-1 involves obligatory extranuclear steps including Raf-1 kinase. EMBO J..

[15] Bode A.M., Dong Z. (2003). Mitogen-activated protein kinase activation in UV-induced signal transduction. Sci. STKE.

[16] Lopez-Camarillo C., Ocampo E.A., Casamichana M.L., Perez-Plasencia C., Alvarez-Sanchez E., Marchat L.A. (2012). Protein kinases and transcription factors activation in response to UV-radiation of skin: Implications for carcinogenesis. Int. J. Mol. Sci..

[17] Fisher G.J., Voorhees J.J. (1998). Molecular Mechanisms of Photoaging and its Prevention by Retinoic Acid: Ultraviolet Irradiation Induces MAP Kinase Signal Transduction Cascades that Induce Ap-1-Regulated Matrix Metalloproteinases that Degrade Human Skin In Vivo. J. Investig. Dermatol. Symp. Proc..

[18] Chrubasik C., Roufogalis B.D., Muller-Ladner U., Chrubasik S. (2008). A systematic review on the *Rosa canina* effect and efficacy profiles. Phytother. Res..

[19] Boskabady M.H., Shafei M.N., Saberi Z., Amini S. (2011). Pharmacological effects of *Rosa Damascena*. Iran. J. Basic Med. Sci..

[20] Fujii T., Ikeda K., Saito M. (2011). Inhibitory effect of rose hip (*Rosa canina* L.) on melanogenesis in mouse melanoma cells and on pigmentation in brown guinea pigs. Biosci. Biotechnol. Biochem..

[21] Jeon H., Kim D.H., Nho Y.H., Park J.E., Kim S.N., Choi E.H. (2016). A Mixture of Extracts of *Kochia scoparia* and *Rosa multiflora* with PPAR α/γ Dual Agonistic Effects Prevents Photoaging in Hairless Mice. Int. J. Mol. Sci..

[22] Pires T.C.S.P., Dias M.I., Barros L., Calhelha R.C., Alves M.J., Oliveira M.B.P.P., Santos-Buelga C., Ferreira I.C.F.R. (2018). Edible flowers as sources of phenolic compounds with bioactive potential. Food Res. Int..

[23] Koczka N., Stefanovits-Banyai E., Ombodi A. (2018). Total Polyphenol Content and Antioxidant Capacity of Rosehips of Some *Rosa* Species. Medicines.

[24] Shin E.J., Han A.R., Lee M.H., Song Y.R., Lee K.M., Nam T.G., Lee P., Lee S.Y., Lim T.G. (2019). Extraction conditions for *Rosa gallica* petal extracts with anti-skin aging activities. Food Sci. Biotechnol..

[25] Shin S.H., Lee J.S., Zhang J.M., Choi S., Boskovic Z.V., Zhao R., Song M., Wang R., Tian J., Lee M.H. (2020). Synthetic lethality by targeting the RUVBL1/2-TTT complex in mTORC1-hyperactive cancer cells. Sci. Adv..

[26] Sano T., Kume T., Fujimura T., Kawada H., Moriwaki S., Takema Y. (2005). The formation of wrinkles caused by transition of keratin intermediate filaments after repetitive UVB exposure. Arch. Derm. Res..

[27] Yamaba H., Haba M., Kunita M., Sakaida T., Tanaka H., Yashiro Y., Nakata S. (2016). Morphological change of skin fibroblasts induced by UV Irradiation is involved in photoaging. Exp. Derm..

[28] Cai Y.Z., Xing J., Sun M., Zhan Z.Q., Corke H. (2005). Phenolic Antioxidants (Hydrolyzable Tannins, Flavonols, and Anthocyanins) Identified by LC-ESI-MS and MALDI-QIT-TOF MS from *Rosa chinensis* flowers. J. Agric. Food Chem..

[29] Barros L., Alves C.T., Duenas M., Silva S., Oliveira R., Carvalho A.M., Henriques M., Santos-Buelga C., Ferreira I.C.F.R. (2013). Characterization of phenolic compounds in wild medicinal flowers from Portugal by HPLC-DAD-ESI/MS and evaluation of antifungal properties. Ind. Crop. Prod..

[30] Kumar N., Bhandari P., Singh B., Bari S.S. (2009). Antioxidant activity and ultra-performance LC-electrospray ionization-quadrupole time-of-flight mass spectrometry for phenolics-based fingerprinting of Rose species: *Rosa damascena*, *Rosa bourboniana* and *Rosa brunonii*. Food Chem. Toxicol..

[31] Ochir S., Nishizawa M., Park B.J., Ishii K., Kanazawa T., Funaki M., Yamagishi T. (2010). Inhibitory effects of *Rosa gallica* on the digestive enzymes. J. Nat. Med..

[32] Qing L.S., Xue Y., Zhang J.G., Zhang Z.F., Liang J., Jiang Y., Liu Y.M., Liao X. (2012). Identification of flavonoid glycosides in *Rosa chinensis* flowers by liquid chromatography-tandem mass spectrometry in combination with ^13^C nuclear magnetic resonance. J. Chromatogr. A.

[33] Ozga J.A., Saeed A., Wismer W., Reinecke D.M. (2007). Characterization of cyanidin- and quercetin-derived flavonoids and other phenolics in mature saskatoon fruits (*Amelanchier alnifolia* Nutt.). J. Agric. Food Chem..

[34] Elejalde-Palmett C., de Bernonville T.D., Glevarec G., Pichon O., Papon N., Courdavault V., St-Pierre B., Giglioli-Guivarc’h N., Lanoue A., Besseau S. (2015). Characterization of a spermidine hydroxycinnamoyltransferase in Malus domestica highlights the evolutionary conservation of trihydroxycinnamoyl spermidines in pollen coat of core Eudicotyledons. J. Exp. Bot..

[35] Tsimogiannis D., Samiotaki M., Panayotou G., Oreopoulou V. (2007). Characterization of flavonoid subgroups and hydroxy substitution by HPLC-MS/MS. Molecules.

[36] Hoyos B., Imam A., Korichneva I., Levi E., Chua R., Hammerling U. (2002). Activation of c-Raf kinase by ultraviolet light. Regulation by retinoids. J. Biol. Chem..

[37] Lee M.H., Nam T.G., Lee I., Shin E.J., Han A.R., Lee P., Lee S.Y., Lim T.G. (2018). Skin anti-inflammatory activity of rose petal extract (*Rosa gallica*) through reduction of MAPK signaling pathway. Food Sci. Nutr..

[38] Shin H.-J., Kim S.-N., Kim J.-K., Lee B.-G., Chang I.-S. (2006). Effect of Green Tea Catechins on the Expression and Activity of MMPs and Type I Procollagen Synthesis in Human Dermal Fibroblasts. J. Soc. Cosmet. Sci. Korea.

[39] Shin E.J., Lee J.S., Hong S., Lim T.G., Byun S. (2019). Quercetin Directly Targets JAK2 and PKCδ and Prevents UV-Induced Photoaging in Human Skin. Int. J. Mol. Sci..

[40] Sudhakaran M., Parra M.R., Stoub H., Gallo K.A., Doseff A.I. (2020). Apigenin by targeting hnRNPA2 sensitizes triple-negative breast cancer spheroids to doxorubicin-induced apoptosis and regulates expression of ABCC4 and ABCG2 drug efflux transporters. Biochem. Pharm..

[41] Lin C.F., Leu Y.L., Al-Suwayeh S.A., Ku M.C., Hwang T.L., Fang J.Y. (2012). Anti-inflammatory activity and percutaneous absorption of quercetin and its polymethoxylated compound and glycosides: The relationships to chemical structures. Eur. J. Pharm. Sci..

[42] Vijayaraj P., Nakagawa H., Yamaki K. (2019). Cyanidin and cyanidin-3-glucoside derived from Vigna unguiculata act as noncompetitive inhibitors of pancreatic lipase. J. Food Biochem..

[43] Hou L., Zhou B., Yang L., Liu Z.L. (2004). Inhibition of human low density lipoprotein oxidation by flavonols and their glycosides. Chem. Phys. Lipids.

[44] Zhao P., Alam M.B., Lee S.H. (2018). Protection of UVB-Induced Photoaging by Fuzhuan-Brick Tea Aqueous Extract via MAPKs/*Nrf2*-Mediated Down-Regulation of MMP-1. Nutrients.

[45] Hwang E., Park S.Y., Lee H.J., Lee T.Y., Sun Z.W., Yi T.H. (2014). Gallic acid regulates skin photoaging in UVB-exposed fibroblast and hairless mice. Phytother. Res..

[46] Green C.L., Khavari P.A. (2004). Targets for molecular therapy of skin cancer. Semin. Cancer Biol..

[47] Khazak V., Astsaturov I., Serebriiskii I.G., Golemis E.A. (2007). Selective Raf inhibition in cancer therapy. Expert Opin. Ther. Targets.

[48] Assi M., Achouri Y., Loriot A., Dauguet N., Dahou H., Baldan J., Libert M., Fain J.S., Guerra C., Bouwens L. (2021). Dynamic Regulation of Expression of KRAS and Its Effectors Determines the Ability to Initiate Tumorigenesis in Pancreatic Acinar Cells. Cancer Res..

[49] Farrand L., Byun S. (2017). Induction of Synthetic Lethality by Natural Compounds Targeting Cancer Signaling. Curr. Pharm. Des..

[50] Brunetti G., Di Rosa G., Scuto M., Leri M., Stefani M., Schmitz-Linneweber C., Calabrese V., Saul N. (2020). Healthspan Maintenance and Prevention of Parkinson’s-like Phenotypes with Hydroxytyrosol and Oleuropein Aglycone in *C. elegans*. Int. J. Mol. Sci..

[51] Calabrese V., Cornelius C., Dinkova-Kostova A.T., Calabrese E.J., Mattson M.P. (2010). Cellular stress responses, the hormesis paradigm, and vitagenes: Novel targets for therapeutic intervention in neurodegenerative disorders. Antioxid. Redox Signal..

[52] Miquel S., Champ C., Day J., Aarts E., Bahr B.A., Bakker M., Banati D., Calabrese V., Cederholm T., Cryan J. (2018). Poor cognitive ageing: Vulnerabilities, mechanisms and the impact of nutritional interventions. Ageing Res. Rev..

[53] Sun Z., Du J., Hwang E., Yi T.H. (2018). Paeonol extracted from *Paeonia suffruticosa* Andr. ameliorated UVB-induced skin photoaging via DLD/Nrf2/ARE and MAPK/AP-1 pathway. Phytother. Res..

[54] Feng R.B., Wang Y., He C., Yang Y., Wan J.B. (2018). Gallic acid, a natural polyphenol, protects against tert-butyl hydroperoxide- induced hepatotoxicity by activating ERK-Nrf2-Keap1-mediated antioxidative response. Food Chem. Toxicol..

[55] Jimenez S., Gascon S., Luquin A., Laguna M., Ancin-Azpilicueta C., Rodriguez-Yoldi M.J. (2016). *Rosa canina* Extracts Have Antiproliferative and Antioxidant Effects on Caco-2 Human Colon Cancer. PLoS ONE.

